# Association between the *TERT* Genetic Polymorphism rs2853676 and Cancer Risk: Meta-Analysis of 76 108 Cases and 134 215 Controls

**DOI:** 10.1371/journal.pone.0128829

**Published:** 2015-06-04

**Authors:** Jin-Lin Cao, Ping Yuan, Abudumailamu Abuduwufuer, Wang Lv, Yun-Hai Yang, Jian Hu

**Affiliations:** Department of Thoracic Surgery, The first Affiliated Hospital, School of Medicine, Zhejiang University, Hangzhou, China; University of Newcastle, UNITED KINGDOM

## Abstract

**Background:**

Several recent studies have identified that the *TERT* genetic polymorphism rs2853676 is associated with cancer risk, but presented inconsistent results. We investigated these inconclusive results by performing a meta-analysis to systematically evaluate the association.

**Methods:**

We conducted a search in PubMed, Google Scholar and ISI Web of Science to select studies on the association between *TERT* rs2853676 and cancer risk. We conducted a stratified analysis using cancer type, ethnicity and source of controls. We calculated the odds ratios (OR) and 95% confidence intervals (CI). Article quality, heterogeneity, sensitivity, publication bias and statistical power were also assessed.

**Results:**

26 articles covering 76 108 cases and 134 215 controls met our inclusion criteria. A significant association between *TERT* rs2853676 allele A and cancer susceptibility was demonstrated under a per-allele risk analysis (OR = 1.08, 95% CI = 1.04-1.13). Stratification analysis revealed an increased cancer risk in subgroups of glioma, lung cancer and ovarian cancer. No significant increase was found in melanoma, breast cancer, pancreatic cancer and colorectal cancer. In a subgroup analysis of lung cancer, a statistically significant increase was only observed in adenocarcinoma. Moreover, a stratified analysis performed for ethnic groups revealed that the significant increase was only observed in Caucasians, whereas a non-significant increase was found in Asians.

**Conclusions:**

This meta-analysis suggests that the *TERT* genetic polymorphism rs2853676 is associated with increased risk of glioma, lung adenocarcinoma and ovarian cancer among Caucasians. Further functional studies are warranted to validate this association and investigate further.

## Introduction

Cancer is a major global public health problem. Fourteen million people were diagnosed with cancer worldwide in 2012. By 2032, the global cancer incidence is predicted to reach to 25 million [1]. In the United States, cancer is the second leading cause of death following heart disease and the leading cause of death among adults aged between 40 and 79 years [2]. Although the causes of cancer are multi-factorial, genetic and environmental factors play an important role in cancer pathogenesis. Recent epidemiological studies have identified several genetic polymorphism loci on chromosome 5p15.33 that are associated with the risk of many types of cancer [3–5]. Chromosome 5p15.33 contains two key genes, *cleft lip and palate transmembrane 1-like* (*CLPTM1L*) and *telomerase reverse transcriptase* (*TERT*).

As the main catalytic subunit of telomerase, *TERT* is essential for the maintenance of telomere DNA length in chromosomes [6]. Telomerase is an RNA-dependent DNA polymerase that synthesizes repetitive DNA (TTAGGG repeats) sequences, which bind abundant specialized proteins onto the chromosome ends [7]. The telomeres prevent coding sequence erosion and protect chromosomes from rearrangements, fusion and genome instability by conducting chromosomal complete replication and regulating gene expression [8]. The expression of telomerase is extremely low in most normal human somatic cells, but is present in over 90% of human malignancies. *In vitro* immortalized cells and the stem cell lines of actively proliferating tissues show a high level of telomerase expression [9, 10]. The activation of telomerase is a vital step during cellular immortalization and the malignant transformation of human cells. This activation requires the *TERT* catalyst [11].

A series of important cancer-related polymorphisms have been reported within the *TERT* gene using a meta-analysis approach and have been identified as contributing to the risk of several cancers, such as the susceptibility to rs2736098 for lung and bladder cancer [12] and that to rs2736100 for lung cancer and glioma [13]. The rs2853676 polymorphism has been mapped to intron 2 of the *TERT* gene, which was implicated in an increased risk of glioma in 2009 [14]. Since then, several studies have assessed the association between the polymorphism and cancer risk, but have presented inconclusive results. We performed a meta-analysis to summarize the available evidence and more precisely characterize the relationship between the *TERT* rs2853676 polymorphism and cancer risk.

## Material and Methods

### Search strategy

According to the Meta-analysis of Observational Studies in Epidemiology guidelines [15], we conducted systematic searches in PubMed, Google Scholar and ISI Web of Science, up to September 20, 2014. We used the systemic literature search terms “*TERT* or rs2853676,” “polymorphism or variant” and “cancer or carcinoma or tumor or neoplasm.” All related reference articles and review articles were searched to identify additional relevant eligible publications. Unpublished data were also obtained from the authors by e-mail.

### Inclusion and exclusion criteria

Identified studies meeting all of the following criteria were included: (1) articles about the *TERT* polymorphism rs2853676 and cancer risk that were published in English; (2) a case-control or case-cohort design addressing race and the numbers of affected and unaffected human control subjects; and (3) sufficient data to calculate an odds ratio (OR) with a 95% confidence interval (CI). The exclusion criteria were: (1) investigations in subjects with family cancer risk; (2) published as an abstract, summary, case report, comment letter, review or editorial; and (3) in overlapped case series, in which case all but the latest or largest study were excluded.

### Data extraction

Data were extracted independently by two investigators, according to the inclusion and exclusion criteria listed above. Discrepancies were resolved by discussion and consensus. We extracted the first author, year of publication, cancer type, patient ethnicity, source of control group (population-based, hospital-based, multiple or nested-in-cohort controls), number of cases and controls, genotyping method, histological subtype, minor allele frequency, genotype and/or per-allele risk OR and 95% CI from each study. The data were extracted separately by population or cancer type, if these were explicitly given. The quality of each study was evaluated using previously published quality assessment criteria [16]. The quality scores of the studies ranged from 0 to 15. Scores ≤ 9 were considered to indicate low quality, while those > 10 were considered to indicate high quality.

### Statistical analysis

Statistical analyses were performed using Stata 12.0 software (Stata Corporation, College Station, Texas). All of the tests were two-sided with a *p*-value. The Hardy-Weinberg equilibrium among the control subjects was assessed with a chi-square test, in which *p* < 0.05 suggested a significant deviation from equilibrium. The OR and 95% CI were calculated to assess the strength of the association between the rs2853676 polymorphism and cancer risk. The significance of the combined OR was determined with a Z test, in which *p* < 0.05 was considered statistically significant. Stratified analyses based on cancer type, ethnicity, histological subtype and source of controls were quantified with ORs and 95% CIs. Ethnicity data sets were categorized as Caucasian, Asian, African or multiple. If a cancer type contained only one data source, it was combined into the “other cancers” group.

The heterogeneity between the studies was calculated by Cochran’s Q-test, in which *p* < 0.10 indicated significant heterogeneity. If the heterogeneity was significant, the random-effects model (DerSimonian and Laird method) was applied [17], otherwise the fixed-effects model was used (Mantel-Haenszel method) [18]. The *I* ^2^ was calculated to quantitatively estimate the heterogeneity, with *I* ^2^ < 25%, *I* ^2^ = 25–75% and *I* ^2^ > 75% representing low, moderate and high heterogeneity, respectively [19].

Sensitivity analyses were performed by sequential removal of each study to assess the stability of the results. Begg’s funnel plots and Egger’s linear regression tests were used to examine the publication bias, in which p < 0.05 indicated statistical significance [20]. Moreover, we estimated the statistical power of each subgroup analysis. Power analyses of the meta-analyses were all conducted using PS (Power and Sample Size Calculations) software version 3.0.5. To guard against Type I errors, α was typically set at 0.05, while β was set at 0.20 to guard against Type II errors, thus a sufficient power of the statistical test would be greater than 80% [21].

## Results

### Eligible studies

**Fig 1:**
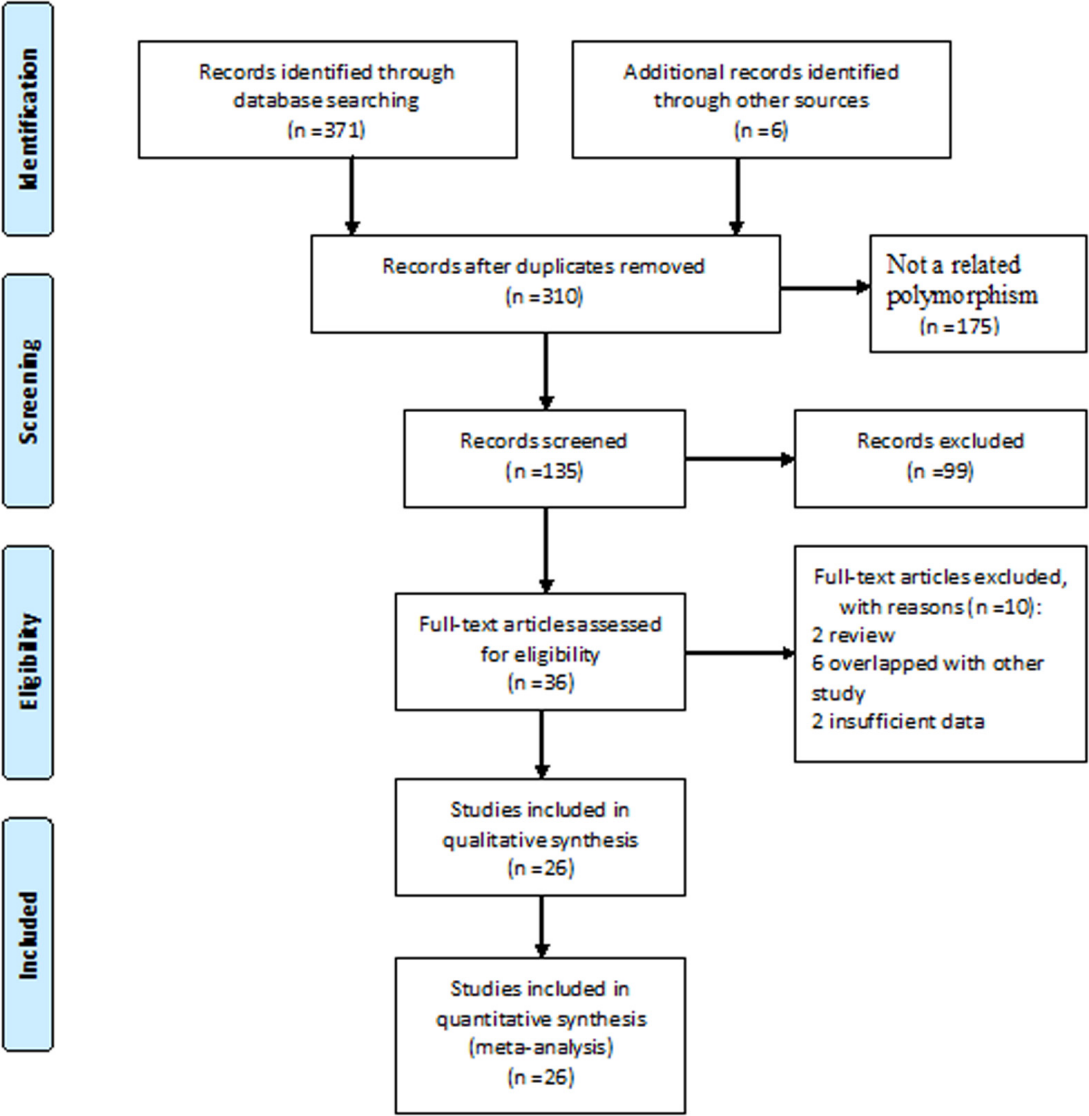
Flow chart of the literature search and selection procedures.

After a comprehensive search, 310 relevant articles were retrieved. Screening of the titles and abstracts excluded 175 articles. Following a full text review and detailed evaluations, 26 articles covering 32 case-control studies with 76 108 cases and 134 215 controls met our inclusion criteria (Fig 1) [4, 14, 22–45]. Among the 32 studies, nine focused on glioma [14, 31, 32, 37, 40], three each on lung cancer [4, 26, 45], breast cancer [22, 39, 43] and melanoma [24, 33, 35], two each on pancreatic cancer [23, 27], ovarian cancer [31, 38] and colorectal cancer [36, 42], and one each on nasopharyngeal cancer [25], endometrial cancer [28], neuroblastoma [34], prostate cancer [41], testicular germ cell cancer [29], acute lymphoblastic leukemia [44], skin squamous cell carcinoma and Basal cell carcinoma [33].

Six studies focused on Asians [25, 26, 31, 37, 44, 45], twenty-four on Caucasians [14, 22–24, 27–30, 32–36, 38, 40–43] and one each on Africans [39] and multiple populations [4]. Ten studies used population-based controls [14, 24, 32, 36, 42, 43, 45], eight used hospital-based controls [14, 25, 31, 33, 35, 37, 40, 44], five used nested-in-cohort controls [22, 28, 33] and nine used multiple controls [4, 23, 26, 27, 29, 30, 38, 39, 41]. The studies used genotyping methods such as Illumina, iPLEX and TaqMan (Table 1). The minor allele frequencies of the control subjects were 25.31% in Caucasians, 17.10% in Asians and 26.4% in Africans.

**Table 1:** Study characteristics of the association between the rs2853676 polymorphism and cancer risk in this meta-analysis.

Author(year)	Ethnicity	Cancer type	Source	Method	Cases/Controls	MAF	OR (95%CI)	Score
Hunter et al.(2007)	Caucasian	Breast	Nested in cohort	Illumina	1145/1142	23.8	0.97(0.81–1.15)	12
Amundadottir et al. (2009) ^a^	Caucasian	Pancreas	Multiple	Illumina	1896/1939	23.8	0.90(0.81–1.00)	11
Falchi et al. (2009)	Caucasian	Melanoma	PB	Illumina	3131/3702	23.8	0.98(0.89–1.07)	13
Shete et al. (French 2009)	Caucasian	Glioma	PB	Illumina	1361/1490	27.0	1.29(1.15–1.44)	14
Shete et al. (German2009)	Caucasian	Glioma	PB	Illumina	498/565	27.0	1.32(1.10–1.59)	14
Shete et al. (Sweden 2009)	Caucasian	Glioma	PB	Illumina	639/760	27.0	1.30(1.10–1.54)	14
Shete et al. (England 2009)	Caucasian	Glioma	PB	Illumina	631/1433	27.0	1.14(0.99–1.32)	14
Shete et al. (America 2009)	Caucasian	Glioma	HB	Illumina	1247/2234	27.0	1.26(1.14–1.41)	12
Bei et al. (2010) ^b^	Asian	Nasopharynx	HB	Illumia	1582/1894	16.3	0.97(0.85–1.11)	12
Hsiung et al. (2010)	Asian	Lung	Multiple	Illumia	2539/2535	17.3	1.08(0.98–1.20)	10
Petersen et al. (2010) ^c^	Caucasian	Pancreas	Multiple	Illumia	1955/1995	23.8	0.96(0.86–1.07)	11
Prescott et al. (2010)^d^	Caucasian	Endometrium	Nested in cohort	Taqman	651/1605	26.1	1.08(0.94–1.25)	12
Turnbull et al. (2010)	Caucasian	Testicular germ cell cancer	Multiple	Illumia	979/4947	27.7	0.75(0.67–0.84)	9
Beesley et al. (2011)^e^	Caucasian	Ovarian	Multiple	iPLEX	990/3687	26.0	1.05(0.93–1.18)	10
Chen et al. (2011)	Asian	Glioma	HB	iPLEX	948/1041	16.0	1.27(1.08–1.49)	11
Egan et al. (2011)	Caucasian	Glioma	PB	Illumia	639/649	28.7	1.22(1.03–1.44)	13
Nan 1 et al. (2011)	Caucasian	Melanoma	Nested in cohort	Taqman	218/840	25.2	1.42(1.13–1.78)	13
Nan 2 et al. (2011)	Caucasian	SCC(skin)	Nested in cohort	Taqman	281/840	25.2	1.10(0.88–1.36)	13
Nan 3 et al. (2011)	Caucasian	BCC(skin)	Nested in cohort	Taqman	284/840	25.2	1.03(0.83–1.29)	13
Wang et al. (2011)	Caucasian	Neuroblastoma	HB	Illumia	2251/6097	27.3	1.03(0.95–1.11)	11
Bodelon et al. (2012)^f^	Caucasian	Melanoma	HB	Illumia	796/770	30.0	1.17(0.84–1.63)	12
Hofer et al. (2012)	Caucasian	Colorectal	PB	Taqman	137/1705	25.0	1.20(0.91–1.58)	14
Liu et al. (2012)	Asian	Glioma	HB	iPLEX	312/311	23.9	0.89(0.69–1.15)	11
Terry et al. (2012)^g^	Caucasian	Ovarian	Multiple	TaqMan	2112/2456	20.0	1.13(1.02–1.25)	10
Zheng et al. (2012)	African	Breast	Multiple	Illumia	1508/1383	22.8	1.02(0.91–1.16)	12
Jin et al. (2013)	Caucasian	Glioma	HB	iPLEX	433/463	15.9	1.56(1.23–1.98)	11
Kote-Jarai et al. (2013)	Caucasian	Prostate	Multiple	Illumia or iPLEX	22301/22320	25.8	1.09(1.05–1.12)	11
Pellatt 1 et al. (2013)	Caucasian	Colorectal	PB	TaqMan	2308/2914	26.0	1.04(0.94–1.14)	13
Pellatt 2 et al. (2013)^h^	Caucasian	Breast	PB	Illumia	3533/4102	19.0	1.23(1.00–1.51)	13
Sheng et al. (2013)	Asian	ALL	HB	Taqman	567/669	16.1	1.19(0.97–1.47)	12
Zhao et al. (2013)	Asian	Lung	PB	SNPscan	782/778	18.0	0.98(0.82–1.18)	14
Park et al. (2014)	Multiple	Lung	Multiple	Illumia	17454/57789	26.4	1.05(1.02–1.09)	11

ALL: Acute lymphoblastic leukemia; SCC: Squamous cell carcinoma; BCC: Basal cell carcinoma; PB: Population based; HB: Hospital based; MAF: Minor allele frequency in control subjects;

^a,c^ Adjusted for study, sex, ancestry and five principal components of population stratification;

^b,f^ Adjusted for age and gender;

^d,e^ Adjust for age and study;

^g^ Adjusted for age (continuous), center, oral contraceptive use, parity, family history of breast or ovarian cancer, and tubal ligation;

^h^ Adjusted for age, study, BMI, vigorous activity in referent year, parity, age at first birth, alcohol consumption, and genetic admixture.

### Meta-analysis results

**Fig 2:**
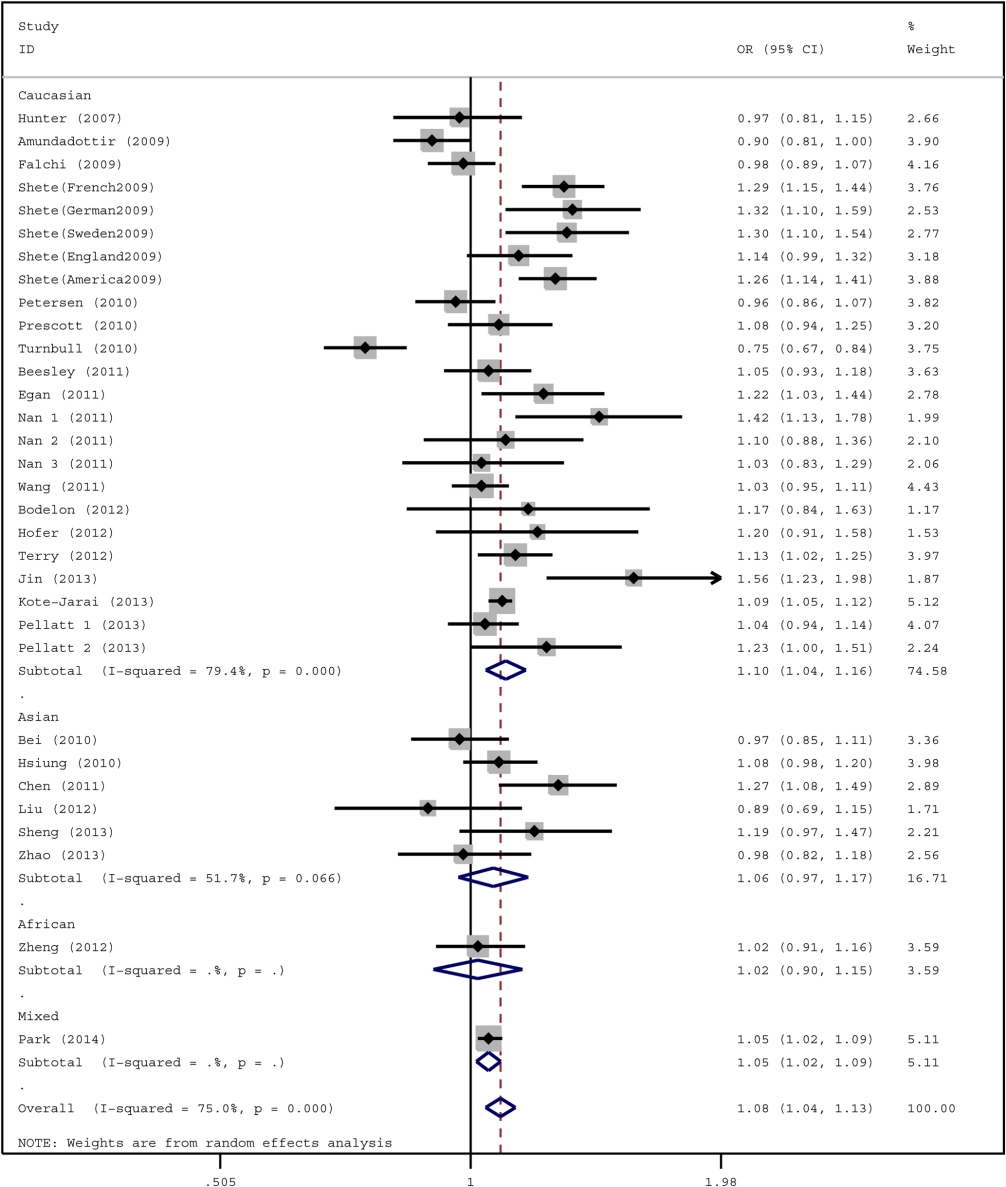
Forest plot of the ORs for the overall cancer risk associated with the rs2853676 polymorphism.

Eight studies were based on adjustment data [23, 25, 27, 28, 30, 35, 38, 43]. These studies had a small effect on the synthesis and did not significantly alter the OR, which agreed with previous results [46, 47]. Based on the data from all 32 studies, we found a significant increased cancer risk for the *TERT* rs2853676 A allele under a per-allele risk analysis (OR = 1.08, 95% CI = 1.04–1.13, *p* < 0.001), with a statistical power of 100%. The results from a random effect model showed significant heterogeneity (*p*_heterogeneity_ < 0.001, *I* ^*2*^ = 75.0%) (Fig 2).

Stratification analysis identified increased cancer risk in subgroups of glioma (per-allele OR = 1.25, 95% CI = 1.19–1.32, *p*_heterogeneity_ = 0.123, *I* ^2^ = 36.9%), lung cancer (per-allele OR = 1.05, 95% CI = 1.02–1.08, *p*_heterogeneity_ = 0.654, *I* ^2^ = 0.0%), ovarian cancer (per-allele OR = 1.10, 95% CI = 1.01–1.18, *p*_heterogeneity_ = 0.358, *I* ^2^ = 0.0%). No significant increase in risk was found in melanoma, breast cancer, pancreatic cancer and colorectal cancer (Table 2). In a subgroup analysis of lung cancer, a statistically significant increase was observed in adenocarcinoma (OR = 1.14, 95% CI = 1.09–1.19, *p*_heterogeneity_ = 0.616, *I* ^2^ = 0.0%) (Table 3). A non-significant difference was found in squamous cell carcinoma (OR = 0.98, 95% CI = 0.92–1.05, *p*_heterogeneity_ = 0.762, *I* ^2^ = 0.0%) and small cell lung carcinoma (OR = 1.05, 95% CI = 0.97–1.15, *p*_heterogeneity_ = 0.826, *I* ^2^ = 0.0%) (data not shown). Moreover, a stratified analysis performed on the ethnicity of the groups revealed that the significant risk was only observed in Caucasians (per-allele OR = 1.10, 95% CI = 1.04–1.16, *p*_heterogeneity_ < 0.001, *I* ^2^ = 79.4%), but a non-significant risk was found in Asians (per-allele OR = 1.06, 95% CI = 0.97–1.17, *p*_heterogeneity_ = 0.066, *I* ^2^ = 51.7%). (Fig 2 and Table 2).

**Table 2:** Stratified analyses of the rs2853676 polymorphism and cancer risk.

Category	No. of		Random effect model		Fixed effect model		*I* ^*2*^ *(%)*	*P*_heterogeneity_	*P*_egger_	Power (%)
	data sets	Cases/Controls	OR(95%CI)	*P*	OR(95%CI)	*P*				
**Total**	32	76108/134215	1.08(1.04–1.13)	<0.001	-	-	75.0	<0.001	0.369	100.0
**Cancer type**										
Glioma	9	6708/8946	-	-	1.25(1.19–1.32)	<0.001	36.9	0.123	0.687	100.0
Lung cancer	3	20075/61102	-	-	1.05(1.02–1.08)	0.002	0.0	0.654	0.821	96.2
Breast cancer	3	6186/6627	-	-	1.04(0.95–1.14)	0.357	38.4	0.197	0.618	24.8
Melanoma	3	4145/5312	1.16(0.89–1.50)	0.265	-	-	78.5	0.010	0.399	99.4
Pancreas	2	3851/3934	-	-	0.93(0.86–1.00)	0.055	0.0	0.405	-	48.0
Ovarian cancer	2	3102/6143	-	-	1.10(1.01–1.18)	0.021	0.0	0.358	-	75.0
Colorectal	2	2445/4619	-	-	1.06(0.96–1.16)	0.238	0.0	0.337	-	30.7
Other cancer	8	28896/38372	1.01(0.92–1.11)	0.794	-	-	83.5	<0.001	0.383	12.4
**Ethnicity**										
Caucasian	24	50416/67815	1.10(1.04–1.16)	0.001	-	-	79.4	<0.001	0.410	100.0
Glioma	7	5448/7594	-	-	1.27(1.20–134)	<0.001	0.0	0.471	0.314	100.0
Breast cancer	2	4678/5244	1.09(0.86–1.37)	0.490	-	-	66.2	0.085	-	69.1
Asian	6	6730/7228	1.06(0.97–1.17)	0.203	-	**-**	51.7	0.066	0.841	45.6
Glioma	2	1260/1352	1.08(0.76–1.53)	0.669	-	-	81.2	0.021	-	19.0
Lung cancer	2	3321/3313	-	-	1.06(0.97–1.15)	0.232	0.0	0.360	-	45.2
African	1	1508/1383	1.02(0.90–1.15)	0.749	-	-	-	-	-	6.1
Multiple	1	17454/57789	1.05(1.02–1.09)	0.004	-	-	-	-	-	94.3
**Source of control**										
Population based	10	13659/18098	1.15(1.06–1.25)	0.001	-	-	66.2	0.002	0.133	100.0
Hospital based	8	8136/13479	1.14(1.03–1.28)	0.016	-	-	73.9	<0.001	0.471	100.0
Nested in cohort	5	2579/3587	-	-	1.09(1.00–1.18)	0.045	44.3	0.127	0.487	54.5
Multiple	9	51734/99051	1.00(0.94–1.07)	0.917	-	-	84.8	<0.001	0.148	5.0

Random effects model was applied when P value for heterogeneity test<0.10, otherwise, fixed effect model was used;

Power calculations assume a = 0.05.

**Table 3:** Stratified analyses of the rs2853676 polymorphism in lung adenocarcinoma.

Author(year)	Ethnicity	Cancer type	Source	Method	Cases/Controls	OR (95%CI)
Hsiung et al. (2010)	Asian	Adenocarcinoma	Multiple	Illumia	1930/2535	1.06(0.95–1.19)
Zhao et al. (2013)	Asian	Adenocarcinoma	PB	SNPscan	359/778	1.04(0.83–1.31)
Park et al. (MEC 2014)	Multiple	Adenocarcinoma	Multiple	Illumia	252/9587	1.19(0.96–1.47)
Park et al. (WHI 2014)	Multiple	Adenocarcinoma	Multiple	Illumia	760/5825	1.11(0.98–1.25)
Park et al. (DeCode Genetics 2014)	Caucasian	Adenocarcinoma	PB	Illumia	346/11225	1.03(0.86–1.22)
Park et al. (Harvard 2014)	Caucasian	Adenocarcinoma	PB	Illumia	488/970	1.26(1.06–1.51)
Park et al. (HGF Germany 2014)	Caucasian	Adenocarcinoma	PB	Illumia	188/479	1.18(0.84–1.67)
Park et al. (IARC GWAS 2014)	Multiple	Adenocarcinoma	Multiple	Illumia	586/3174	1.25(1.08–1.44)
Park et al. (MDACC 2014)	Caucasian	Adenocarcinoma	Multiple	Illumia	619/1133	1.09(0.93–1.28)
Park et al. (NCI GWAS 2014)	Multiple	Adenocarcinoma	Multiple	Illumia	1836/5686	1.19(1.09–1.29)
Park et al. (SLRI/ Toronto 2014)	Caucasian	Adenocarcinoma	PB	Illumia	89/488	1.07(0.76–1.51)

PB: Population based; HB: Hospital based;

The overall OR = 1.14, 95% CI: 1.09–1.19, *P* <0.001; *P*_heterogeneity_ = 0.616, *I* ^2^ = 0.0%.

A stratified analysis by source of controls indicated a significantly increased risk associated with population-based, hospital-based, and nested-in-cohort controls, with ORs of 1.15 (95% CI = 1.06–1.25), 1.14 (95% CI = 1.03–1.28), and 1.09 (95% CI = 1.00–1.18), respectively. No significant increase was found in multiple-source controls. A stratified analysis by cancer type was also performed in Caucasians and Asians. The results for glioma and breast cancer were the same in Caucasians as in the overall population, but a non-significant increase in risk was found for glioma and lung cancer in Asians (Table 2).

### Heterogeneity test and sensitivity analyses

Significant heterogeneity existed, mainly in all cancer and the subgroups of ethnicity, population-based controls, hospital-based controls and multiple-source controls. However, most of the heterogeneity disappeared, except melanoma and “other cancers,” in the analysis of cancer type subgroups (Table 2).

**Fig 3:**
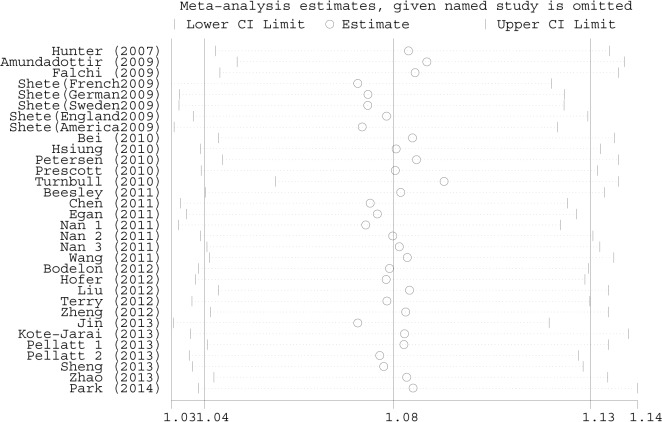
Sensitivity analysis of the overall ORs. The results were calculated by omitting each eligible study. Meta-analysis random-effects estimates (exponential form) were used.

A sensitivity analysis was conducted to assess the influence of each study, by sequential omission of each eligible study. The results showed that the significance of the OR was not affected by any single study (Fig 3). In addition, after the removal of a study that resulted in a departure from the Hardy-Weinberg equilibrium, no significant alteration was found in the OR.

### Publication bias

**Fig 4:**
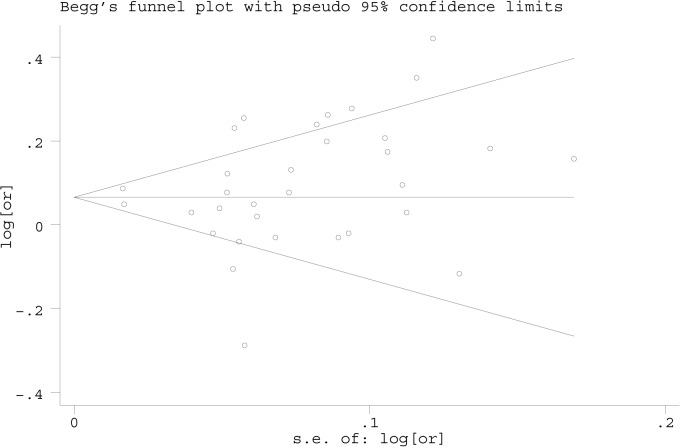
Funnel plot analysis to detect publication bias for the rs2853676 polymorphism in the 32 data sets.

Publication bias was assessed with Begg’s funnel plots and Egger’s test. The shapes of the funnel plots did not show any evidence of publication bias (Fig 4). No significant publication bias was found by Egger’s test in the overall or subgroup analyses (Table 2).

## Discussion

The *TERT* gene is the main catalytic subunit of telomerase, which is encoded by a single-copy gene, mapped on chromosome 5p15.33 and contains 16 exons and 15 introns spanning about 35 kb [48]. The gene consists of three distinct structural domains: an RNA-binding domain, a reverse transcriptase domain and a carboxy- terminal extension, which is thought to represent the putative thumb domain of *TERT* [49]. A high level of *TERT* expression is involved in a variety of human malignancies. *TERT* may play an important role in the pathogenesis of cancer [6, 9, 50]. The *TERT* gene sequence has been proposed as a general mechanism affecting individual susceptibility to cancer risk [4, 5, 47]. A growing number of epidemiological studies have been conducted in response, which have provided evidence that *TERT* polymorphisms contribute to cancer development [4, 5].

The polymorphism rs2853676 is located in intron 2 of the *TERT* gene. The association between this polymorphism and cancer risk has been assessed in several studies, which showed inconclusive results. Only one meta-analysis demonstrated strong evidence that rs2853676 increased the risk of central nervous system tumors, but the evidence for the risk of lung cancer was weak [51]. A recent study from the Population Architecture using the Genomics and Epidemiology and Transdisciplinary Research in Cancer of the Lung consortia identified that rs2853676 was associated with an increased risk of lung adenocarcinoma [4]. Our meta-analysis suggested that the *TERT* genetic polymorphism rs2853676 allele A increased several cancer risk, based on 76 108 cases and 134 215 controls. The association mainly existed in the Caucasian population, especially for glioma, lung cancer and ovarian cancer. No significant association was found in melanoma, breast cancer, pancreatic cancer or colorectal cancer. In a subgroup analysis of lung cancer, a statistically significant association was only observed in adenocarcinoma. Interestingly, the Asian population showed no significant result in any type of cancer. Notably, one study described an increased risk for prostate cancer [41], whereas another described a reduced risk for testicular germ cell cancer [29]. Further studies are required to validate these associations in urogenital system tumors.

The heterogeneity between our studies was significantly reduced in the analysis of the cancer type subgroups, indicating that the effect of *TERT* polymorphisms may be modified by tumor origin. The effect may be cancer-type specific and play a different role in the etiology of different tumors [47]. However, the exact functional mechanisms underlying the association between the rs2853676 polymorphism and cancer remains unclear. Several studies have suggested that telomere length alters cancer risk [5, 33, 52]. However, this alteration has not been observed in rs2853676 [28, 38, 43], except by Melin et al., who detected potential relevance at higher ages in a small sample [53]. The other plausible mechanisms underlying the association between the rs2853676 polymorphism and cancer risk may be attributable to environmental risk factors or genetic background. The modification of the *TERT* function is likely to also play an important role. A high linkage disequilibrium with other nearby biologically potential functional polymorphisms or disease-causing mutations may also exist. In National Cancer Institute controls, rs2853676 was in modest linkage disequilibrium (r^2^ = 0.25, D’ = 0.82) with rs2736100 and showed a similar pattern of association with lung adenocarcinoma (OR = 1.16, *p* = 3.44×10^–4^) [54]. A recent Japanese study also identified that *TERT* rs2853677 (European ancestry: r^2^ = 0.59) was associated with lung adenocarcinoma (*p* = 3.1×10^–40^) [55]. Park and collaborators speculated that the association between rs2853676 and adenocarcinoma may be influenced by rs2736100 and rs2853677 [4].

Several limitations in this meta-analysis should be addressed. First, our meta-analysis only presented limited studies that were available to adjust the estimates, and more individual data would be required to draw a more precise conclusion. Second, gene-gene and gene-environment interactions may have influenced our results, as cancer is mainly caused by genetic and environmental factors. However, no appropriate information was available to test this. Third, not all of the authors of the included studies agreed to provide their data and exact genotype data were reported in a minority of the studies. The analysis was therefore only conducted with an additive model (per-allele risk analysis). Fourth, in this meta-analysis, the power of several subgroup results was < 80%, indicating that additional high-level studies are still needed.

In conclusion, this meta-analysis suggests that the *TERT* genetic polymorphism rs2853676 is associated with an increased risk of glioma, lung adenocarcinoma and ovarian cancer among Caucasians, suggesting that the association may be cancer-type and ethnically specific. To validate this association and investigate our findings further, functional studies are warranted.

## Supporting Information

S1 File PRISMA 2009 Flow Diagram. A list of full-text excluded articles.
(DOC)

S2 File PRISMA Checklist. Meta-analysis on Genetic Association Studies Checklist.
(DOCX)

S1 Table Genotype frequencies and per-allele OR (95%CI) of each data set enrolled. (DOC)
