# Supplementary material for: Association between the TERT Genetic Polymorphism rs2853676 and Cancer Risk: Meta-Analysis of 76 108 Cases and 134 215 Controls
Source: PLoS One. 2015 Jun 4;10(6):e0128829. doi: 10.1371/journal.pone.0128829 (PMC4456375; doi:10.1371/journal.pone.0128829)
Supplement: S1 File — A list of full-text excluded articles. (DOC) [file pone.0128829.s001.doc]

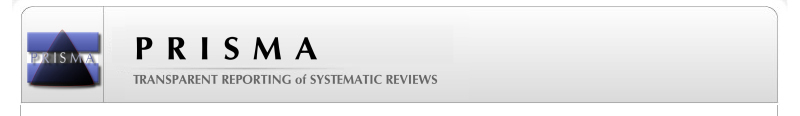
**A List of Full-text Excluded Articles**

**Screening**

**Included**

**Eligibility**

**Identification**

Records identified through database searching
(n =371)

Additional records identified through other sources
(n =6)

Records after duplicates removed
(n =310)

Records screened
(n =135)

Records excluded
(n =99)

Full-text articles assessed for eligibility
(n =36)

Full-text articles excluded, with reasons (n =10):

2 review

6 overlapped with other study

2 insufficient data

Studies included in qualitative synthesis
(n =26)

Studies included in quantitative synthesis (meta-analysis)
(n =26)

Not a related polymorphism
 (n =175)
