# Supplementary material for: Association between the TERT Genetic Polymorphism rs2853676 and Cancer Risk: Meta-Analysis of 76 108 Cases and 134 215 Controls
Source: PLoS One. 2015 Jun 4;10(6):e0128829. doi: 10.1371/journal.pone.0128829 (PMC4456375; doi:10.1371/journal.pone.0128829)
Supplement: S1 Table — (DOC) [file pone.0128829.s003.doc]

**S1 Table.** Genotype frequencies and per-allele OR (95%CI) of each data set enrolled.

| **Author(year)** | **Genotype of Cases/ Controls** | | | | | **OR (95%CI)**^a^ | **OR (95%CI)**^b^ | | **Note** |
| --- | --- | --- | --- | --- | --- | --- | --- | --- | --- |
|  | **AA** | | | **AG** | **GG** |  |  |  |  |
| Hunter (2007)[22] | |  |  | |  | 0.97(0.81-1.15) |  | GWAS discovery phase | |
| Amundadottir (2009)[23] |  | | |  |  | 0.90(0.81-1.00) |  | GWAS discovery phase | |
| Falchi (2009)[24] |  | | |  |  | 0.98(0.89-1.07) |  |  | |
| Shete (French 2009)[14] | 164/127 | | | 623/622 | 574/741 |  | 1.29(1.15-1.44) | GWAS discovery phase | |
| Shete (German2009)[14] | 52/45 | | | 217/209 | 229/311 |  | 1.32(1.10-1.59) | GWAS discovery phase | |
| Shete (Sweden 2009)[14] | 51/45 | | | 264/269 | 324/446 |  | 1.30(1.10-1.54) | GWAS discovery phase | |
| Shete (England 2009)[14] | 67/113 | | | 263/596 | 301/724 |  | 1.14(0.99-1.32) | GWAS discovery phase | |
| Shete (America 2009)[14] | 109/149 | | | 568/894 | 570/1191 |  | 1.26(1.14-1.41) | GWAS discovery phase | |
| Bei (2010)[25] |  | | |  |  | 0.97(0.85-1.11) |  | GWAS discovery +replication phase | |
| Hsiung (2010)[26] | 93/92 | | | 751/693 | 1695/1750 |  | 1.08(0.98-1.20) | GWAS discovery +replication phase | |
| Petersen (2010)[27] |  | | |  |  | 0.96(0.86-1.07) |  | GWAS discovery phase | |
| Prescott (2010)[28] |  | | |  |  | 1.08(0.94-1.25) |  | Females only | |
| Turnbull (2010)[29] |  | | |  |  | 0.75(0.67-0.84) |  | GWAS discovery phase | |
| Beesley (2011)[30] |  | | |  |  | 1.05(0.93-1.18) |  |  | |
| Chen (2011)[31] | 42/16 | | | 286/302 | 620/723 |  | 1.27(1.08-1.49) |  | |
| Egan (2011)[32] |  | | |  |  | 1.22(1.03-1.44) |  |  | |
| Nan 1 (2011)[33] | 22/54 | | | 97/316 | 99/470 |  | 1.42(1.13-1.78) | Females only | |
| Nan 2 (2011)[33] | 25/54 | | | 102/316 | 154/470 |  | 1.10(0.88-1.36) | Females only | |
| Nan 3 (2011)[33] | 19/54 | | | 109/316 | 156/470 |  | 1.03(0.83-1.29) | Females only | |
| Wang (2011)[34] |  | | |  |  | 1.03(0.95-1.11) |  | GWAS discovery phase | |
| Bodelon (2012)[35] |  | | |  |  | 1.17(0.84-1.63) |  |  | |
| Hofer (2012)[36] | 12/117 | | | 54/616 | 71/972 |  | 1.20(0.91-1.58) |  | |
| Liu (2012)[37] | 177/183 | | | 108/108 | 27/20 |  | 0.89(0.69-1.15) |  | |
| Terry (2012)[38] |  | | |  |  | 1.13(1.02-1.25) |  |  | |
| Zheng (2012)[39] | 83/83 | | | 534/464 | 891/836 |  | 1.02(0.91-1.16) |  | |
| Jin (2013)[40] | 23/17 | | | 151/113 | 259/333 |  | 1.56(1.23-1.98) |  | |
| Kote-Jarai (2013)[41] |  | | |  |  | 1.09(1.05-1.12) |  | GWAS discovery +replication phase | |
| Pellatt 1 (2013)[42] | 160/217 | | | 916/1082 | 1232/1615 |  | 1.04(0.94-1.14) |  | |
| Pellatt 2 (2013)[43] | 206/186 | | |  |  | 1.23(1.00-1.51) |  |  | |
| Sheng (2013)[44] | 7/15 | | | 198/186 | 362/468 |  | 1.19(0.97-1.47) |  | |
| Zhao (2013)[45] | 21/26 | | | 231/224 | 530/528 |  | 0.98(0.82-1.18) |  | |
| Park (2014)[4] |  | | |  |  | 1.05(1.02-1.09) |  | GWAS discovery phase | |

^a^ Crude OR extracted from citation;

^b^ ORs were calculated in additive model;

Nan1-3 represented studies for melanoma, squamous cell and basal cell carcinomas, respectively;

Pellatt 1-2 represented studies for colorectal and breast cancer, respectively
